# Influence of Confinement Due to COVID-19 on Physical Activity and Mediterranean Diet Adherence and Its Relationship with Self-Esteem in Pre-Adolescent Students

**DOI:** 10.3390/children8100848

**Published:** 2021-09-25

**Authors:** Gracia Cristina Villodres, Laura García-Pérez, Juan Miguel Corpas, José Joaquín Muros

**Affiliations:** Department of Didactics of Corporal Expression, University of Granada, 18071 Granada, Spain; cristina1998@correo.ugr.es (G.C.V.); arualgp10@correo.ugr.es (L.G.-P.); jumicoru15@correo.ugr.es (J.M.C.)

**Keywords:** COVID-19, socioeconomic status, physical activity, diet, self-esteem

## Abstract

Regular physical activity and good adherence to the Mediterranean diet are important for improving physical and mental health. Confinement due to the COVID-19 pandemic has led to a lack of exercise and poor nutrition. Preadolescent mental health, specifically regarding self-esteem, may have been affected. This is particularly relevant between the ages of 10 and 14. The influence of total confinement due to COVID-19 on physical activity and Mediterranean diet adherence, and its relationship with self-esteem was studied in third-year primary school students and first-year secondary school students in the provinces of Granada and Malaga, Spain. Validated questionnaires were administered to evaluate physical activity (PAQ-C), Mediterranean diet adherence (KIDMED) and self-esteem (Rosenberg Scale). In the same way, the FAS III test was used to evaluate socioeconomic status and an ad-hoc questionnaire was developed to collect sociodemographic data and evaluate screen time. Data were analyzed using IBM SPSS 25.0 statistical software. During the period of total confinement, statistically significant differences were found between examined variables, according to sex, school year, school type, socioeconomic status and whether or not the participant had contracted COVID-19. These data were compared with those collected during a previous time period. Those with a low socioeconomic status and girls were most affected.

## 1. Introduction

In December 2019, the SARS-CoV-2 coronavirus was identified in Wuhan, China [1]. This virus causes the disease known colloquially and worldwide as COVID-19 [2] and is characterized by severe respiratory syndrome [3]. Due to the rate and extent of its spread, a state of urgent international health emergency and global pandemic was declared [4].

Spain and more than 100 countries announced a state of alarm, leading to a period of uncertainty and insecurity [5]. This resulted in unexpected changes to the lifestyle of citizens, decreasing all types of socialization [1]. Children all over the world were affected by confinement measures, concretely, home confinement, social distancing, and measures related with the educational environment and the consequent adaptations required at home [6,7]. Children belonging to low socioeconomic status families have been most adversely affected [8,9,10].

Although public health measures and government restrictions helped to reduce infection rates, further significant negative effects on aspects of physical and mental health are expected in the future [11,12] in both the short and long term [13]. This is likely to be mainly due to decreased daily physical activity (PA) [14], loss of opportunities to be active [8] and limitations to accessing quality food [15].

During the period of total confinement due to COVID-19, it was difficult to avoid engaging in sedentary habits, such as spending lots of time in front of screens [16]. Prior to this period, most of the PA engaged in by young people was derived through walking to school, physical education classes, break time, after-school activities, social activities, etc. With confinement, these opportunities decreased, especially in individuals with limited space to move around [8].

At the same time, consumption of calorie dense foods increased due to anxiety, stress and boredom [12]. Likewise, parents reported feeding their children more frequently than usual [17] as a means to avoiding boredom or stress [18]. Parents more often turned to foods considered “comfort foods” which are rich in sugars, salt, fats, etc. [19]. Further, low socioeconomic status families have presented comparably greater food insecurity than their richer counterparts and, therefore, have been shown to have consumed lower quality food during the period of total confinement [10].

A decline in self-esteem has been observed within preadolescents due to the greater frequency with which they engaged in these bad habits during confinement [20]. Alterations in other mental health factors, such as anxiety and depression, have also been observed [21].

Prior to the pandemic, more than 81% of the population aged between 11 and 17 years were already inactive worldwide [22]. In Spain, 76.6% of this age group failed to meet the PA levels recommended by WHO, with this rate being even higher in girls (83.8%) than boys (69.8%) [23]. Further, 64.1% of the Spanish population failed to follow a Mediterranean diet (MD) in 2019 [24], whilst between 10% and 20% of the global population had a mental health disorder [25]. Moreover, 73.9% of the Spanish population aged between 1 and 14 years already spent more than one hour per day in front of the screen on weekdays, with this figure rising to 82.6% on weekends and also being higher in disadvantaged groups [26].

Previous results indicate that dietary changes in a given context, time and space, and in unexpected situations such as confinement, can negatively impact cardiometabolic health and increase BMI [27,28,29], as well as increase the risk of cardiovascular disease, obesity, mental health disorders, etc. [30]. Similarly, physical inactivity is directly related with greater rates of overweight, obesity and type 2 diabetes, and reduced quality of life [31,32,33,34]. Likewise, obesity, a sedentary lifestyle and poor academic performance has been associated with low self-esteem [35].

Uncertainty about the personal and global effects of COVID-19 is a cause of great concern, particularly, with regards to the potential impact of lockdown on aforementioned percentages [12].

A MD is a diet that is low in saturated fat and characterized by a high consumption of plant-based foods (fruits, vegetables, whole grains, nuts and seeds), olive oil, dairy products (cheese and yoghurt) and eggs, low-moderate amounts of fish and poultry, and a low consumption of red meat and wine [36]. Adequate PA levels and avoidance of sedentary habits are positively related with following a healthy diet, such as a MD [37]. The opposite is true in stressful situations [38]. Both not only reduce the risk of respiratory infection [39] and inflammation [40], and prevent many chronic conditions that increase the risk of severe COVID-19 infection [41,42]. Preliminary results indicate that the characteristic components of the MD may favor the pro-inflammatory actions of platelet-activating factor (PAF) [43]. A healthy diet containing PAF inhibitors may target both inflammation and thrombosis and prevent the deleterious effects of COVID-19 [44]. Better adherence to a Mediterranean diet may, therefore, be associated with a lower risk of COVID-19, as indicated by the results of different studies [45,46].

Also, both are beneficial when it comes to reducing symptoms of anxiety and depression and raising self-esteem [47]. In this regard, it is essential to consider self-esteem from early ages in order to increase health-related quality of life [48].

Consequently, the scientific community should strive to examine the habits engaged in during confinement and analyze their influence on the physical and mental state of children and adolescents. Furthermore, knowledge of these effects is of great interest to present and future pandemic strategies [49,50,51]. This will be especially important for identifying vulnerable groups and target good responses to these situations [52].

Thus, the aim of the present research is to study the influence of the period of total confinement due to COVID-19 on PA engagement and MD adherence, and its relationship with self-esteem in pre-adolescent students. It also aims to determine the influence of socioeconomic status and gender.

## 2. Materials and Methods

### 2.1. Design and Participants

A non-experimental, descriptive, cross-sectional, analytical, case-control and correlational study was designed.

Nine hundred and seventeen pre-adolescent participants formed the total sample. From these, 18 questionnaires were discarded due to having been incorrectly filled in. For this reason, 899 students formed the final sample. Students were undertaking third year primary school education (PE) (N = 257) or first year secondary school education (SE) (N = 642). Students came from state and mixed funding schools in the provinces of Granada and Malaga, Spain.

The final sample was made up by 415 boys (46.2%) and 484 girls (53.8%).

Sampling was not random, with participants being selected according to convenience. All students participated voluntarily, with the prior consent of their parents or legal guardians. Schools and the Parents’ Associations authorized the study.

### 2.2. Instruments

The PA questionnaire for children and pre-adolescents aged between 8 and 14 years denominated the physical activity questionnaire for children (PAQ-C) [53] was used to evaluate physical activity engagement. The present study used a translated version that has been cross-culturally adapted and validated in Spanish [54]. It has been shown to be valid and reliable, producing an intraclass correlation coefficient (ICC) greater than 0.73 and an internal consistency of α = 0.86. It consists of 10 items designed to measure moderate and vigorous PA engagement over a period of seven days. Nine of these items are scored from 1 to 5. Overall test scores are obtained from the mean of these nine items. The tenth item provides a validity check as respondents report whether they have experienced any personal impediment to carrying out their usual PA during the 7 days considered by the questionnaire. None of the participants marked this item affirmatively.

The KIDMED test [55] was used to evaluate MD adherence. The latest updated version adapted into Spanish was used [56]. It consists of 16 items designed to measure levels of MD adherence in children and adolescents. Items must be answered affirmatively (yes) or negatively (no). Four of the items are negatively framed, with positive responses being scored −1. In contrast, positive responses to positively framed items are scored +1. Negative responses have a score of 0. Thus, the total index ranges from −4 to 12. From this MD adherence is categorized along the following continuum: Poor quality (≤3); Needs improvement (4–7); Optimal quality (≥8).

For the evaluation of personal self-esteem, a version of the Rosenberg self-esteem scale [57] which has been adapted, translated and validated in Spanish [58] was used. This has been shown to be valid and reliable, with an internal consistency of α = 0.80. It consists of 10 items which are designed to evaluate the level of personal self-esteem. The first five are positively framed. Responses are listed from A to D and are scored from 4 to 1. The remaining five are negatively framed. In this case, responses are listed from A to D and are scored from 1 to 4. This serves to control for effects of self-administered acquiescence. Total scores are determined by summing together all of the scores obtained for each individual item. The maximum possible score is 40 and the minimum is 10. Scores are categorized according to the following: Low i.e., significant self-esteem problems (10–25); Medium i.e., improvements are desirable (26–29); High i.e., normal self-esteem (30–40).

In order to evaluate socioeconomic status, the Spanish translation of the family affluence scale (FAS III) [59] was used. This is the most recent version of the FAS [60]. This scale has been shown to be valid and reliable, with an internal consistency of between α= 0.76 and 0.91. It is made up of 6 items designed to evaluate family purchasing power based on material goods. All items are scored between 0 and 3. Total scores are obtained by calculating the sum of individual scores. The maximum possible score is 13 and the minimum is zero [61]. Scores are categorized according to: Low (0–2); Medium (3–5); High (≥6).

An ad-hoc and bespoke questionnaire was developed to collect sociodemographic data: sex, date of birth, province of residence, height and weight. BMI was calculated from height and weight [BMI = weight [kg]/(height [m])^2^]. Age and sex standardized classifications of normal weight, overweight and obese were calculated, according to the international cut-points defined by Cole et al. [62] for boys and girls aged between 2 and 18 years. Screen time was also evaluated. Two items were added to examine the number of hours spent daily on screen-based leisure activities (watching television, playing video games, using a mobile phone, using a computer, etc.) on both weekdays and weekends. Another item asked whether or not participants had been infected with COVID-19.

### 2.3. Procedure

Prior to data collection, two information packs were prepared to secure informed consent. These included information on all of the characteristics and conditions of the study. The first was addressed to the directors of the educational centers. The second was addressed to the parents or legal guardians of the students. The purpose of this was to ensure that procedures were conducted in an appropriate legislative and ethical manner.

Given the different situations experienced by educational centers due to COVID-19, questionnaires could be completed in two different ways. Firstly, students could complete questionnaires individually during school hours in the presence of a researcher. Secondly, students could complete questionnaires individually via Google forms. The link was sent to students by their school headteacher or other teachers at the participating centers. 

In order to evaluate the influence of COVID-19 on examined variables, participants completed the same questionnaire twice. The first time, they were asked to consider the period of total confinement or lockdown (15 March 2020–21 June 2020). The second time, they were asked to consider a time period prior to the onset of COVID-19. Both questionnaires were completed on the same day. This method has been used in other previous studies [63].

Data collection was carried out during the months of February, March and April 2021.

### 2.4. Data Analysis

Data were analyzed using IBM SPSS 25.0 statistical software. Qualitative variables (sex, school year, school type, BMI, socioeconomic status and whether or not the participant had been infected with COVID-19) are presented as percentages. Quantitative variables are presented as means and standard deviations. Sample distribution was analyzed using the Kolmogorov–Smirnov test. After verifying that data followed a non-normal distribution, the Wilcoxon test was used to compare two related samples, Mann-Whitney U test to compare two unrelated groups and Kruskal Wallis test to compare more than two groups, where relevant. Correlations were performed using Spearman’s test. Significance was set at *p* = 0.05.

## 3. Results

Table 1 shows the characteristics of the sample during the period of total confinement, according to sex, school year, school type, socioeconomic status, BMI and whether or not they had been infected with COVID-19.

During the period of total confinement due to COVID-19, students reported spending an average of 5.77 ± 3.23 h per day in front of a screen. Mean scores were obtained of 2.33 ± 0.69 on the PAQ-C, 6.04 ± 2.73 on the KIDMED and 29.67 ± 6.28 on the Rosenberg scale.

Of the total number of students, 46.2% were boys and 53.7% were girls. Statistically significant differences were found in relation to screen time (*p* = 0.023) and self-esteem (*p* = 0.001). Boys were observed to engage in more screen time (6.00 ± 9.41 vs. 5.58 ± 3.23) and have higher self-esteem test than girls (30.60 ± 5.47 vs. 28.88 ± 6.80).

On the other hand, statistically significant differences were not found with regards to PA engagement and MD adherence.

Fewer participating students were undertaking the third year of PE (28.6%) than the first year of SE (71.4%). Statistically significant differences were found in relation to screen time (*p* = 0.000). SE students spent more time in front of the screen than PE students (6.06 ± 3.16 vs. 5.04 ± 3.30). Likewise, PE students were observed to engage in more PA than SE students (2.48 ± 0.68 vs. 2.27 ± 0.68), adherence more stringently to a MD (6.59 ± 2.67 vs. 5.81 ± 2.73) and have higher self-esteem (32.40 ± 5.30 vs. 28.58 ± 6.31). These differences were also statistically significant (*p* = 0.000).

In addition, 30.3% of participating students attended mixed funding schools, while 69.7% attended state schools. Statistically significant differences were observed in relation to screen time, with this being higher in state school students (6.04 ± 3.25 vs. 5.15 ± 3.08). Likewise, students belonging to mixed funding schools reported higher PA engagement (2.40 ± 0.65 vs. 2.30 ± 0.70), MD adherence (6.90 ± 2.50 vs. 5.66 ± 2.75) and self-esteem (30.92 ± 5.94 vs. 29.14 ± 6.35). These differences were statistically significant (*p* = 0.036, *p* = 0.000 and *p* = 0.000, respectively).

With regards to socioeconomic status, most participants belonged to families with a high socioeconomic status (84%). Statistically significant differences were observed in screen time (*p* = 0.010), observing that students with a lower socioeconomic status spent more hours in front of screens compared with medium and high socioeconomic status students (8.39 ± .41 vs. 6.22 ± 3.41 and 5.77 ± 3.23). Likewise, significant differences were found in MD adherence (*p* = 0.000) and self-esteem (0.027). Students with a lower socioeconomic status reported lower scores for MD adherence (6.19 ± 2.69 vs. 5.36 ± 2.84 vs. 3.87 ± 2.50) and self-esteem (29.94 ± 6.16 vs. 28.93 ± 6.70 vs. 28.21 ± 6.70). 

Further, significant differences were not found in relation to PA. However, a tendency towards decreased PA engagement was observed as socioeconomic status decreased (2.34 ± 0.67 vs. 2.29 ± 0.76 and 2.14 ± 0.70).

Table 2 presents sample characteristics and compares, both overall and in relation to sex, data collected during the period of total confinement with the pre-confinement period.

Statistically significant differences were observed in both sexes in relation to the variables of screen time (*p* = 0.000), PA (*p* = 0.000) and self-esteem (*p* = 0.000).

Both boys and girls spent more hours in front of screens during total confinement compared to the pre-confinement period (5.77 ± 3.23 vs. 3.26 ± 2.09).

Likewise, both sexes reported less PA (2.33 ± 0.69 vs. 2.87 ± 0.72) during total confinement and scores referring to self-esteem were lower in the period corresponding to total confinement period than the pre-confinement period (29.67 ± 6.28 vs. 30.43 ± 6.30). Statistically significant differences were not observed with regards to MD adherence.

Data was analyzed in a number of ways in order to observe differences between the period of total confinement and the pre-confinement period, in light of all remaining study variables.

Statistically significant differences according to school year, school type and socioeconomic status were found at the level of *p* = 0.000 in relation to screen time, PA and self-esteem.

With regards to the MD adherence variable, statistically significant differences (*p* = 0.027) were only found in students undertaking the first year of SE, with higher scores emerging in relation to total confinement than pre-confinement (5.81 ± 2.73 vs. 5.65 ± 2.87).

Table 3 presents the sex-adjusted correlation coefficients produced between the study variables.

A positive correlation was observed between PA and MD adherence (r = 0.275; *p* = 0.000), PA and self-esteem (r = 0.091; *p* = 0.007), and PA and socioeconomic status (r = 0.111; *p* = 0.001). In contrast, PA correlated negatively with BMI (r = −0.072; *p* = 0.031), screen time (r = −0.143; *p* = 0.000) and age (r = −0.191; *p* = 0.000).

Next, a positive correlation was found between MD and socioeconomic status (r = 0.145; *p* = 0.000), and MD adherence and self-esteem (r = 0.237; *p* = 0.000). Likewise, MD adherence correlated negatively with screen time (r = −0.282; *p* = 0.000) and age (r = −0.147; *p* = 0.000).

On the other hand, a negative correlation was observed between self-esteem and screen time (r = −0.143; *p* = 0.000), as well as between self-esteem and age (r = −0.261; *p* = 0.000).

Further, BMI was negatively correlated with socioeconomic status (r = −0.171; *p* = 0.000) and positively correlated with screen time (r = 0.139; *p* = 0.000).

Finally, screen time correlated positively with age (r = 0.231; *p* = 0.000).

## 4. Discussion

The change of circumstances imposed by confinement due to COVID-19 has limited opportunities to engage in physical activity and consume to high-quality food in the pre-adolescent population. This could negatively influence their physical and mental health. The aim of the present research was to study the influence of the period of total confinement due to COVID-19 on PA engagement and MD adherence and examine its relationship with self-esteem in pre-adolescent students. In addition, in accordance with existing literature, family socioeconomic status and sex were considered as relevant factors for examination.

Findings of the present study established that pre-adolescent boys and girls exhibited worse adherence to healthy lifestyle habits during the period of total confinement due to COVID-19 relative to a prior time-period. This led to negative effects on self-esteem, especially within individuals with lower socioeconomic status and females.

Firstly, a decrease in PA was observed during confinement in both boys and girls. Other studies, such as that conducted by Medrano et al. [64], also observed overall PA engagement to decrease during confinement in 291 Spanish preadolescents. These authors also observed that screen time increased to an average of 6 h a day, with increases being particularly prominent in boys, as was the case in the present study.

PA decreased with age in the present study. Thus, SE students engaged in less PA than PE students during the period of total confinement. Dunton et al. [65] reported similar findings in American schoolchildren and preadolescents. By way of explanation, they concluded that older students spent more time than younger students using mobile phones and computers with an Internet connection, playing video games, listening to music or chatting during their leisure time. For the same reason, screen time was also seen to increase with age. This agrees with present findings which show that SE students report spending more time in front of a screen than PE students.

According to the literature, regular PA engagement goes hand in hand with a healthy diet. In this regard, Pietrobelli et al. [29] observed PA engagement to decrease in Italian children and adolescents during total confinement. In turn, adherence to a healthy diet decreased, as shown by a positive correlation between PA and MD adherence in the present study.

Significant differences were not found in adherence to the MD between the confinement period and the pre-confinement period for any of the study dimensions. However, despite the negative correlation between MD and age, students undertaking SE were observed to improve their MD adherence during confinement. Ruiz-Roso et al. [66] obtained similar results, reporting that students aged between 13 and 14 years increased their intake of vegetables, fruits, legumes, etc. to a greater extent than their younger counterparts during confinement with respect to a period prior to confinement. It was also found that cooking at home with one’s parents reduced boredom and the frequency with which young people went out to fast food restaurants with friends. All of the aforementioned may have a greater impact on older preadolescents.

Teixeira et al. [67] also observed the same positive correlation between MD adherence and socioeconomic status as that found in the present research. They concluded that total confinement predominantly affected the eating habits of children and adolescents belonging to families with a low socioeconomic status, as they consumed fewer fresh fruits and vegetables than other children. Indeed, the price of high-quality food went up during confinement due to limitations in purchasing low-cost food. As a consequence, only families with high purchasing power were able to access better quality foodstuffs [68].

With regards to that discussed above, children belonging to low socioeconomic status families engaged in less PA in the present study. A negative correlation between PA and socioeconomic status was also observed. During the period of confinement, opportunities to engage in physical activity were reduced [8] due to the closure of schools, sports clubs and public spaces, etc. Thus, children who did not reside in houses with large outdoor spaces or yards saw their access to PA curtailed [69].

Similarly, boys and girls belonging to low socioeconomic status families spent more hours in front of a screen. Within this population group, PA in their leisure time was replaced with time in front of a screen. The latter habit is often accompanied by the consumption of energy-dense foods and snacks that are rich in fats and sugars. Thus, following an unhealthy diet and reducing PA engagement results in an unhealthy rise in BMI [70,71]. In a similar sense, this may explain the positive correlation seen in the present study between BMI and screen time. It is also likely to explain the negative correlation of PA and MD with screen time, and that seen between BMI and socioeconomic status.

Further, those with low socioeconomic status reported having the lowest self-esteem. Families with higher incomes a more able to work from home than those with lower incomes. The latter tend to occupy more unstable job positions [72]. As a result, high socioeconomic status families have stated feeling a stronger bond [73] due to spending more time on parental care duties. This is positively reflected in the self-esteem levels of their children [74].

Another important aspect to consider relates to the present finding of higher PA engagement, MD adherence and self-esteem in mixed funding schools compared to state schools, alongside lower levels of screen time. Previous studies [75] have concluded that Spanish parents with a higher socioeconomic status tend to enroll their children in mixed funding schools as they more highly value this type of education. In addition, such families are able to cover mandatory fees and, in so doing, bypass selective admission processes [76]. In this sense, the fact that those enrolled at mixed funding schools have better outcomes than those attending state schools, may be explained by present outcomes pertaining to the sub-group with a high socioeconomic status of higher PA engagement, MD adherence and self-esteem, and lower screen time. 

With regards to self-esteem, it is well accepted that children aged between 12 and 14 years are more susceptible to the influence of personal beliefs and show continuous changes in self-esteem [77]. In the present study, a negative correlation between self-esteem and age was observed. In addition, SE students obtained significantly lower self-esteem scores than PE students. Research such as that conducted by Gittins and Hunt [78] found that self-esteem levels begin to drop from the age of 12. This occurs due to the emergence of self-criticism and self-evaluations of self-worth. This has been associated with the development of negative mood states, specifically, within this age group.

A positive correlation was obtained between PA, MD adherence and self-esteem, whilst a negative correlation was produced between screen time and self-esteem, regardless of sex. Despite this, girls were observed to report lower PA engagement and self-esteem than boys during the period of total confinement. Previous research has argued that access barriers cause women to engage in alarmingly less PA than men [79]. Such barriers appear to persist during confinement.

Similarly, both sexes increased the number of hours they spent viewing screens during total confinement. Furthermore, in accordance with Medrano et al. [63] boys spent more time in front of screens, both before and during confinement, than girls. However, a negative effect was seen in girls. Girls belonging to the generation under study often use mobile devices linked with the social network Instagram. They report following profiles focused on appearance, which indirectly leads to eating disorders and body dissatisfaction. This leads to a negative influence on self-esteem [80].

There is, therefore, an additional social pressure on women. This exposes them to the negative effects of social ideals, while, at the same time, being subjected to access barriers to sport [79]. All of the aforementioned leads to detrimental effects such as increased mental distress, with this being associated with low MD adherence in adulthood [81]. Consequently, situations that promote detrimental aspects in women’s physical and mental health should be avoided.

In general, self-esteem ratings were lower during confinement with respect to a prior time-period. In the present study, it was noteworthy that students who had been infected with COVID-19 presented lower levels of self-esteem during the period of total confinement than the other participants. Other studies have highlighted self-esteem as a predictor of anxiety symptoms. These symptoms are greater in this group due to fear of possible infection and its consequences [82]. Greenberg et al.’s terror management theory [83] outlines the way in which human awareness of the possibility of death (in this case caused by COVID-19) conflicts with human tendencies for survival. This has negative implications for different factors related with mental health.

## 5. Conclusions

Students of pre-adolescent age were less likely to engage in healthy habits during the period of total confinement, negatively affecting their self-esteem. 

On the one hand, low socioeconomic status negatively influenced PA engagement due to the limitations imposed on appropriate adequate spaces. On the other hand, low socioeconomic status had a negative influence on MD adherence due to the limited access to fresh and high-quality food experienced during confinement. Implications of this are reflected in the findings pertaining to self-esteem. Generally speaking, self-esteem decreased amongst those with low socioeconomic status. 

For this reason, vulnerable groups must be identified in order to attend to and address their needs, enabling an appropriate response to other similar situations to be taken. Access to PA and quality food should be facilitated, as well as access to mental health services. This is largely catered for by healthcare services which are not covered by social security in Spain.

As a further aspect, the influence of total confinement due to COVID-19 on study variables was analyzed according to sex. It was determined that girls engaged in less PA and had lower self-esteem than boys. This premise was found to already exist prior to confinement, although the strength of relationships increased during total confinement.

Thus, education must work to urgently dismantle stereotypes and prejudices against women that obstruct women’s access to sports. In addition, it is important to promote the eradication of beauty ideals created by society as they lead women to feel obliged to meet unrealistic and unhealthy standards. This has a negative influence on their eating habits and self-esteem.

In short, scientific and educational communities must study, outline and develop future strategies which encourage appropriate responses to uncertain situations, such as that generated by the current COVID-19 pandemic, or any other similar pandemic that could occur in the future. Above all, vulnerable populations that are most exposed to its negative effects should be the focus of attention.

## 6. Limitations and Future Perspectives

The present study has a number of limitations that should be considered. Potential alternatives are suggested in order for future research to provide more reliable and meaningful results. 

Firstly, questionnaires were used to measure study variables that increase measurement error. However, all of the tools employed (PAQ-C, KIDMED and Rosenberg scale) have shown sufficient reliability and validity for use with this type of population. Little impact is therefore foreseen on final conclusions. Alternatively, accessible and practical technological instruments such as pulsometers or accelerometers could be used. Such instruments record more accurate data on PA frequency and intensity over given time-points.

Secondly, simultaneous collection of data pertaining to time-points both before and after confinement may reduce data accuracy. Nonetheless, other published research has employed the same method [63]. Alternatively, data could be collected in real time, at two different time-points (before and during confinement). This would ensure more accurate data collection; however, it is a difficult issue to address given that the emergence and spread of COVID-19 and the associated total confinement were unforeseen.

With regards to future perspectives, it would be interesting to create, implement and study the effects of didactic resources encouraging engagement in the healthy habits examined here. In addition, such resources should primarily target the most vulnerable groups. It is envisaged that outputs of the present work will also be useful for approaching possible future confinements.

## Figures and Tables

**Table 1:** Sample characteristics during the period of total confinement, according to sex, school year, school type, socioeconomic status, BMI and whether or not they had been infected with COVID-19.

Characteristics		N	%	ST	*p*	PA	*p*	MD	*p*	SE	*p*
TOTAL		899	100%	5.77 ± 3.23		2.33 ± 0.69		6.04 ± 2.73		29.67 ± 6.28	
SEX	Boys	415	46.2%	6.00 ± 9.41	0.023	2.37 ± 0.722	0.238	6.17 ± 2.48	0.139	30.60 ± 5.47	0.001
	Girls	484	53.7%	5.58 ± 3.23		2.29 ± 0.65		5.92 ± 2.93		28.88 ± 6.80	
SCHOOL YEAR	Primary	257	28.6%	5.04 ± 3.30	0.000	2.48 ± 0.68	0.000	6.59 ± 2.67	0.000	32.40 ± 5.30	0.000
	Secondary	642	71.4%	6.06 ± 3.16		2.27 ± 0.68		5.81 ± 2.73		28.58 ± 6.31	
SCHOOL TYPE	MFS	272	30.3%	5.15 ± 3.08	0.000	2.40 ± 0.65	0.036	6.90 ± 2.50	0.000	30.92 ± 5.94	0.000
	State	627	69.7%	6.04 ± 3.25		2.30 ± 0.70		5.66 ± 2.75		29.14 ± 6.35	
SS	Low	15	1.7%	8.39 ± .41	0.010	2.14 ± 0.70	0.551	3.87 ± 2.50	0.000	28.21 ± 6.70	0.027
	Medium	129	14.3%	6.22 ± 3.41		2.29 ± 0.76		5.36 ± 2.84		28.93 ± 6.70	
	High	755	84%	5.77 ± 3.23		2.34 ± 0.67		6.19 ± 2.69		29.94 ± 6.16	
BMI	Normal	690	76.8%	5.71 ± 3.17	0.505	2.32 ± 0.69	0.072	6.06 ± 2.69	0.890	29.53 ± 6.44	0.470
	Overweight	176	19.6%	6.06 ± 3.45		2.31 ± 0.69		5.93 ± 2.90		30.14 ± 5.83	
	Obesity	33	3.7%	5.48 ± 3.14		2.56 ± 0.69		6.03 ± 2.80		30.18 ± 5.06	
COVID	Yes	105	88.3%	5.98 ± 3.17	0.411	2.42 ± 0.57	0.104	5.85 ± 2.70	0.777	28.15 ± 6.07	0.006
	No	794	11.7%	5.74 ± 3.24		2.32 ± 0.70		6.06 ± 2.74		29.88 ± 6.28	

Notes. ST: Screen time, PA: Physical activity; MD: Mediterranean diet; MFS: Mixed funding schools; SS: Socioeconomic status; BMI: Body mass index; SE: Self esteem.

**Table 2:** Differences in sample characteristics between the period of total confinement and pre-confinement, according to sex.

Characteristics			ST	*p*	PA	*p*	MD	*p*	SE	*p*
TOTAL	BEFORE		3.26 ± 2.09	0.000	2.87 ± 0.72	0.000	5.98 ± 2.85	0.273	30.43 ± 6.30	0.000
	AFTER		5.77 ± 3.23		2.33 ± 0.69		6.04 ± 2.73		29.67 ± 6.28	
SEX	Boys	BEFORE	3.32 ± 2.04	0.000	3.00 ± 0.73	0.000	6.10 ± 2.63	0.455	31.08 ± 5.71	0.000
		AFTER	6.00 ± 3.22		2.37 ± 0.72		6.17 ± 2.48		30.6 ± 5.47	
	Girls	BEFORE	3.20 ± 2.13	0.000	2.78 ± 0.70	0.000	5.87 ± 3.02	0.427	29.87 ± 6.72	0.000
		AFTER	5.58 ± 3.23		2.29 ± 0.65		5.92 ± 2.93		28.88 ± 6.80	

Notes. ST: Screen time, PA: Physical activity; MD: Mediterranean diet; SE: Self esteem.

**Table 3:** Sex-adjusted correlations between study variables.

	SS	ST	PA	MD	SE	AGE
BMI	−0.171 **	0.139 **	−0.072 *	−0.042	−0.043	0.256 **
SS	.	−0.059	0.111 **	0.145 **	0.063	−0.146 **
ST		.	−0.14 3**	−0.282 **	−0.143 **	0.231 **
PA			.	0.275 **	0.091 **	−0.191 **
MD				.	0.237 **	−0.147 **
SE					.	−0.261 **

Notes. BMI: Body mass index; SS: Socioeconomic status; ST: Screen time; PA: Physical activity; MD: Mediterranean diet; SE: Self esteem. * *p* ˂ 0.05, ** *p* ˂ 0.01.

## References

[1] Orientaciones Para El Público. https://www.who.int/es/emergencies/diseases/novel-coronavirus-2019/advice-for-public.

[2] Gómez C.C., Rodríguez Ó.P., Torné M.L., Santaolalla C.E., Jiménez J.F.M., Fernández J.G., Perales J.M.C., Heili-Frades S.B., Monreal M.F., de Andrés Nilsson J.M. (2020). Recomendaciones de consenso respecto al soporte respiratorio no invasivo en el paciente adulto con insuficiencia respiratoria aguda secundaria a infección por SARS-CoV-2. Med. Intensiva.

[3] Alonso-Martínez A.M., Ramírez-Vélez R., García-Alonso Y., Izquierdo M., García-Hermoso A. (2021). Physical activity, sedentary behavior, sleep and self-regulation in spanish preschoolers during the COVID-19 lockdown. Int. J. Environ. Res. Public Health.

[4] COVID-19: Cronología de la Actuación de la OMS. https://www.who.int/es/news/item/27-04-2020-who-timeline---COVID-19.

[5] Arafat S.M.Y., Kar S.K., Marthoenis M., Sharma P., Hoque Apu E., Kabir R. (2020). Psychological underpinning of panic buying during pandemic (COVID-19). Psychiatry Res..

[6] Bahn G.H. (2020). Coronavirus disease 2019, school closures, and children’s mental health. J. Korean Acad. Child Adolesc. Psychiatry.

[7] Szabo T.G., Richling S., Embry D.D., Biglan A., Wilson K.G. (2020). From helpless to hero: Promoting values-based behavior and positive family interaction in the midst of COVID-19. Behav. Anal. Pract..

[8] Guan H., Okely A.D., Aguilar-Farias N., Del Pozo Cruz B., Draper C.E., El Hamdouchi A., Florindo A.A., Jáuregui A., Katzmarzyk P.T., Kontsevaya A. (2020). Promoting healthy movement behaviours among children during the COVID-19 pandemic. Lancet Child Adolesc. Health.

[9] Ghosh R., Dubey M.J., Chatterjee S., Dubey S. (2020). Impact of COVID-19 on children: Special focus on the psychosocial aspect. Minerva Pediatr..

[10] Van Lancker W., Parolin Z. (2020). COVID-19, school closures, and child poverty: A social crisis in the making. Lancet Public Health.

[11] Xiang Y.-T., Yang Y., Li W., Zhang L., Zhang Q., Cheung T., Ng C.H. (2020). Timely mental health care for the 2019 novel coronavirus outbreak is urgently needed. Lancet Psychiatry.

[12] Brooks S.K., Webster R., ESmith L., Woodland L., Wessely S., Greenberg N., Rubin G.J. (2020). The psychological impact of quarantine and how to reduce it: Rapid review of the evidence. Lancet.

[13] Fiorillo A., Gorwood P. (2020). The consequences of the COVID-19 pandemic on mental health and implications for clinical practice. Eur. Psychiatry.

[14] Hossain M.M., Sultana A., Purohit N. (2020). Mental Health Outcomes of Quarantine and Isolation for Infection Prevention: A Systematic Umbrella Review of the Global Evidence.

[15] BDA Eating Well during Coronavirus/COVID-19. https://www.bda.uk.com/resource/eating-well-during-coronavirus-covid-19.html.

[16] Schmidt S.C.E., Anedda B., Burchartz A., Eichsteller A., Kolb S., Nigg C., Niessner C., Oriwol D., Worth A., Woll A. (2020). Physical activity and screen time of children and adolescents before and during the COVID-19 lockdown in Germany: A natural experiment. Sci. Rep..

[17] Cluver L., Lachman J.M., Sherr L., Wessels I., Krug E., Rakotomalala S., Blight S., Hillis S., Bachman G., Green O. (2020). Parenting in a time of COVID-19. Lancet.

[18] Snuggs S., Houston-Price C., Harvey K. (2019). Development of a parental feeding goal measure: The family mealtime goals questionnaire. Front. Psychol..

[19] Yılmaz C., Gökmen V. (2020). Neuroactive compounds in foods: Occurrence, mechanism and potential health effects. Food Res. Int..

[20] Adıbelli D., Sümen A. (2020). The effect of the coronavirus (COVID-19) pandemic on health-related quality of life in children. Child. Youth Serv. Rev..

[21] (2020). Interpersonally-Based Fears during the COVID-19 Pandemic: Reflections on the Fear of Missing out and the Fear of Not Mattering Constructs|Clinical Neuropsychiatry. https://www.clinicalneuropsychiatry.org/download/interpersonally-based-fears-during-the-covid-19-pandemic-reflections-on-the-fear-of-missing-out-and-the-fear-of-not-mattering-constructs/.

[22] Physical Activity. https://www.who.int/news-room/fact-sheets/detail/physical-activity.

[23] El Ejercicio Físico En Los Adolescentes, Datos y Estadísticas. https://www.epdata.es/datos/ejercicio-fisico-adolescentes-datos-estadisticas/479.

[24] Estudio PASOS Resultados Finales Gasol Foundation. https://www.gasolfoundation.org/es/estudio-pasos/.

[25] Adolescent Mental Health. https://www.who.int/news-room/fact-sheets/detail/adolescent-mental-health.

[26] Ministerio de Sanidad, Consumo y Bienestar Social-Portal Estadístico del SNS-Encuesta Nacional de Salud de España 2017. https://www.mscbs.gob.es/estadEstudios/estadisticas/encuestaNacional/encuesta2017.htm.

[27] King A.J., Burke L.M., Halson S.L., Hawley J.A. (2020). The challenge of maintaining metabolic health during a global. Pandemic. Sports Med..

[28] Robinson E., Boyland E., Chisholm A., Harrold J., Maloney N.G., Marty L., Mead B.R., Noonan R., Hardman C.A. (2021). Obesity, eating behavior and physical activity during COVID-19 lockdown: A study of UK adults. Appetite.

[29] Pietrobelli A., Pecoraro L., Ferruzzi A., Heo M., Faith M., Zoller T., Antoniazzi F., Piacentini G., Fearnbach S.N., Heymsfield S.B. (2020). Effects of COVID-19 lockdown on lifestyle behaviors in children with obesity living in Verona, Italy: A longitudinal study. Obesity.

[30] Chandrasekaran B., Ganesan T.B. (2021). Sedentarism and chronic disease risk in COVID 19 lockdown—A scoping review. Scott. Med. J..

[31] Cole T.J., Lobstein T. (2012). Extended international (IOTF) body mass index cut-offs for thinness, overweight and obesity. Pediatr. Obes..

[32] Biswas A., Oh P.I., Faulkner G.E., Bajaj R.R., Silver M.A., Mitchell M.S., Alter D.A. (2015). Sedentary time and its association with risk for disease incidence, mortality, and hospitalization in adults. Ann. Intern. Med..

[33] Estruch R., Ros E. (2020). The role of the Mediterranean diet on weight loss and obesity-related diseases. Rev. Endocr. Metab. Disord..

[34] Rosado J.R., Fernández Á.I., López J.M. (2020). Evaluación de la práctica de actividad física, la adherencia a la dieta y el comportamiento y su relación con la calidad de vida en estudiantes de Educación Primaria (Physical activity patterns, nutritional habits, and behaviours and their relation with. Retos.

[35] McClure A.C., Tanski S.E., Kingsbury J., Gerrard M., Sargent J.D. (2010). Characteristics associated with low self-esteem among US adolescents. Acad. Pediatr..

[36] Willett W.C., Sacks F., Trichopoulou A., Drescher G., Ferro-Luzzi A., Helsing E., Trichopoulos D. (1995). Mediterranean diet pyramid: A cultural model for healthy eating. Am. J. Clin. Nutr..

[37] Muros J.J., Cofre-Bolados C., Arriscado D., Zurita F., Knox E. (2017). Mediterranean diet adherence is associated with lifestyle, physical fitness, and mental wellness among 10-y-olds in Chile. Nutrition.

[38] Tate E.B., Spruijt-Metz D., Pickering T.A., Pentz M.A. (2015). Two facets of stress and indirect effects on child diet through emotion-driven eating. Eat. Behav..

[39] Martin S.A., Pence B.D., Woods J.A. (2009). Exercise and respiratory tract viral infections. Exerc. Sport Sci. Rev..

[40] Suzuki K. (2019). Chronic Inflammation as an immunological abnormality and effectiveness of exercise. Biomolecules.

[41] Jordan R.E., Adab P., Cheng K.K. (2020). COVID-19: Risk factors for severe disease and death. BMJ.

[42] Lesser I.A., Nienhuis C.P. (2020). The impact of COVID-19 on physical activity behavior and well-being of canadians. Int. J. Environ. Res. Public Health.

[43] Nomikos T., Fragopoulou E., Antonopoulou S., Panagiotakos D.B. (2018). Mediterranean diet and platelet-activating factor; a systematic review. Clin. Biochem..

[44] Detopoulou P., Demopoulos C.A., Antonopoulou S. (2021). Micronutrients, phytochemicals and mediterranean diet: A potential protective role against COVID-19 through modulation of PAF actions and metabolism. Nutrients.

[45] Perez-Araluce R., Martinez-Gonzalez M.A., Fernández-Lázaro C.I., Bes-Rastrollo M., Gea A., Carlos S. (2021). Mediterranean diet and the risk of COVID-19 in the ‘seguimiento universidad de navarra’ cohort. Clin. Nutr. Edinb. Scotl..

[46] Perez-Araluce R., Martinez-Gonzalez M., Fernández-Lázaro C., Bes-Rastrollo M., Gea A., Carlos S. (2021). Mediterranean diet and SARS-COV-2 infection: Is there any association? A proof-of-concept study. Nutrients.

[47] Biddle S.J.H., Asare M. (2011). Physical activity and mental health in children and adolescents: A review of reviews. Br. J. Sports Med..

[48] Martinsen K.D., Rasmussen L.-M.P., Wentzel-Larsen T., Holen S., Sund A.M., Pedersen M.L., Løvaas M.E.S., Patras J., Adolfsen F., Neumer S.-P. (2021). Change in quality of life and self-esteem in a randomized controlled CBT study for anxious and sad children: Can targeting anxious and depressive symptoms improve functional domains in schoolchildren?. BMC Psychol..

[49] Sara Latham B.A., Jamie Lamson M.P.H., Stephanie Williams R.R.T., Michelle N., Eakin P. (2020). Maintaining emotional well-being during the COVID-19 pandemic: A resource for your patients. Chronic Obs. Pulm. Dis. COPD Found..

[50] Psychological Intervention Measures during the COVID-19 Pandemic|Clinical Neuropsychiatry. https://www.clinicalneuropsychiatry.org/download/psychological-intervention-measures-during-the-covid-19-pandemic/.

[51] Xiang M., Zhang Z., Kuwahara K. (2020). Impact of COVID-19 pandemic on children and adolescents’ lifestyle behavior larger than expected. Prog. Cardiovasc. Dis..

[52] Woolf H.R., Fair M., King S.B., Dunn C.G., Kaczynski A.T. (2020). Exploring dietary behavior differences among children by race/ethnicity and socioeconomic status. J. Sch. Health.

[53] Crocker P.R.E., Bailey D.A., Faulkner R.A., Kowalski K.C., McGRATH R. (1997). Measuring general levels of physical activity: Preliminary evidence for the Physical activity questionnaire for older children. Med. Sci. Sports Exerc..

[54] Manchola-González J., Bagur-Calafat C., Girabent-Farrés M. Fiabilidad de la versión española del cuestionario de actividad física PAQ-C/reliability of the spanish version of questionnaire of physical activity PAQ-C. Rev. Int. Med. Cienc. Act. Física Deporte 2017, 139–152..

[55] Serra-Majem L., Ribas L., Ngo J., Ortega R.M., García A., Pérez-Rodrigo C., Aranceta J. (2004). Food, youth and the Mediterranean diet in Spain. Development of KIDMED, Mediterranean Diet Quality Index in children and adolescents. Public Health Nutr..

[56] Altavilla C., Comeche Guijarro J.M., Comino I., Caballero P. (2020). El índice de Calidad de la Dieta Mediterránea en la Infancia y la Adolescencia (KIDMED)Propuesta de Actualización Para Países Hispanohablantes. Spanish Update of the KIDMED Questionnaire, a MEDITERRANEAN Diet Quality Index in Children and Adolescents..

[57] Rosenberg M. (2015). Society and the Adolescent Self-Image.

[58] Atienza F.L. (2000). Análisis de la dimensionalidad de la escala de autoestima de Rosenberg en una muestra de adolescentes valencianos. Rev. Psicol. Univ. Tarracon..

[59] Hobza V., Hamrik Z., Bucksch J., De Clercq B. (2017). The family affluence scale as an indicator for socioeconomic status: Validation on regional income differences in the czech republic. Int. J. Environ. Res. Public Health.

[60] Currie C.E., Elton R.A., Todd J., Platt S. (1997). Indicators of socioeconomic status for adolescents: The WHO health behaviour in school-aged children survey. Health Educ. Res..

[61] Moreno Maldonado C., Moreno Rodríguez M.D.C., Rivera de los Santos F.J. (2016). Indicadores Para Detectar y Evaluar el Impacto de las Desigualdades Socioeconómicas en los Estilos de Vida y la Salud de Los Adolescentes Españoles. https://idus.us.es/handle/11441/64495.

[62] Cole T.J., Bellizzi M.C., Flegal K.M., Dietz W.H. (2000). Establishing a standard definition for child overweight and obesity worldwide: International survey. BMJ.

[63] Ammar A., Brach M., Trabelsi K., Chtourou H., Boukhris O., Masmoudi L., Bouaziz B., Bentlage E., How D., Ahmed M. (2020). Effects of COVID-19 Home Confinement on Physical Activity and Eating Behaviour Preliminary Results of the ECLB-COVID19 International Online-Survey. https://www.medrxiv.org/content/10.1101/2020.05.04.20072447v1.

[64] Medrano M., Cadenas-Sanchez C., Oses M., Arenaza L., Amasene M., Labayen I. (2021). Changes in lifestyle behaviours during the COVID-19 confinement in Spanish children: A longitudinal analysis from the MUGI project. Pediatr. Obes..

[65] Dunton G.F., Do B., Wang S.D. (2020). Early effects of the COVID-19 pandemic on physical activity and sedentary behavior in children living in the U.S. BMC Public Health.

[66] Ruiz-Roso M.B., de Carvalho Padilha P., Mantilla-Escalante D.C., Ulloa N., Brun P., Acevedo-Correa D., Arantes Ferreira Peres W., Martorell M., Aires M.T., de Oliveira Cardoso L. (2020). Covid-19 confinement and changes of adolescent’s dietary trends in Italy, Spain, Chile, Colombia and Brazil. Nutrients.

[67] Teixeira M.T., Vitorino R.S., Silva J.H., da Raposo L.M., Aquino L.A., de Ribas S.A. (2021). Eating habits of children and adolescents during the COVID-19 pandemic: The impact of social isolation. J. Hum. Nutr. Diet..

[68] Altieri M.A., Nicholls C.I. (2020). Agroecology and the emergence of a post COVID-19 agriculture. Agric. Hum. Values..

[69] Pombo A., Luz C., Rodrigues L.P., Ferreira C., Cordovil R. (2020). Correlates of children’s physical activity during the COVID-19 confinement in Portugal. Public Health.

[70] Avery A., Anderson C., McCullough F. (2017). Associations between children’s diet quality and watching television during meal or snack consumption: A systematic review. Matern. Child Nutr..

[71] Deschasaux-Tanguy M., Druesne-Pecollo N., Esseddik Y., de Edelenyi F.S., Allès B., Andreeva V.A., Baudry J., Charreire H., Deschamps V., Egnell M. (2021). Diet and physical activity during the coronavirus disease 2019 (COVID-19) lockdown (March–May 2020): Results from the French NutriNet-Santé cohort study. Am. J. Clin. Nutr..

[72] McKibbin W., Fernando R. (2021). The global macroeconomic impacts of COVID-19: Seven scenarios. Asian Econ. Pap..

[73] (2020). Quarantine Quality Time: 4 In 5 Parents Say Coronavirus Lockdown Has Brought Family Closer Together. https://www.studyfinds.org/quarantine-quality-time-4-in-5-parents-say-coronavirus-lockdown-has-brought-family-closer-together/.

[74] Doi S., Isumi A., Fujiwara T. (2020). The association between parental involvement behavior and self-esteem among adolescents living in poverty: Results from the K-CHILD study. Int. J. Environ. Res. Public Health.

[75] Rogero García J., Andrés Candelas M. (2016). Representaciones Sociales de los Padres y Madres Sobre la Educación Pública y Privada en España. http://dspace2.ti.uam.es:8080/xmlui/handle/10486/677365.

[76] Santos H., Elacqua G. (2016). Segregación Socioeconómica Escolar en Chile: Elección de la Escuela por los Padres y un Análisis Contrafactual Teórico. https://repositorio.cepal.org/handle/11362/40396.

[77] Shahar G., Henrich C.C. (2010). Do depressive symptoms erode self-esteem in early adolescence?. Self Identity.

[78] Gittins C.B., Hunt C. (2020). Self-criticism and self-esteem in early adolescence: Do they predict depression?. PLoS ONE.

[79] González N.F., Rivas A.D. (2018). Actividad física y ejercicio en la mujer. Rev. Colomb. Cardiol..

[80] Vall-Roqué H., Andrés A., Saldaña C. (2021). The impact of COVID-19 lockdown on social network sites use, body image disturbances and self-esteem among adolescent and young women. Prog. Neuropsychopharmacol. Biol. Psychiatry..

[81] Salvatore F.P., Relja A., Filipčić I.Š., Polašek O., Kolčić I. (2019). Mediterranean diet and mental distress: “10,001 Dalmatians” study. Br. Food J..

[82] Rossi A., Panzeri A., Pietrabissa G., Manzoni G.M., Castelnuovo G., Mannarini S. (2020). The anxiety-buffer hypothesis in the time of COVID-19: When self-esteem protects from the impact of loneliness and fear on anxiety and depression. Front. Psychol..

[83] Greenberg J., Pyszczynski T., Solomon S., Baumeister R.F. (1986). The Causes and Consequences of a Need for Self-Esteem: A Terror Management Theory. Public Self and Private Self.

